# MutT homolog 1 counteracts the effect of anti-neoplastic treatments in adult and pediatric glioblastoma cells

**DOI:** 10.18632/oncotarget.25547

**Published:** 2018-06-08

**Authors:** Ziv Versano, Eitan Shany, Shany Freedman, Liron Tuval-Kochen, Moshe Leitner, Shoshana Paglin, Amos Toren, Michal Yalon

**Affiliations:** ^1^ Pediatric Hemato-Oncology, Edmond and Lilly Safra Children's Hospital and Cancer Research Center, Sheba Medical Center, Ramat Gan 52621, Israel; ^2^ Sackler School of Medicine, Tel Aviv University, Tel Aviv 69978, Israel; ^3^ The Talpiot Medical Leadership Program, Chaim Sheba Medical Center, Ramat Gan 52621, Israel

**Keywords:** MutT homolog 1, Nudix hydrolase, adult and pediatric glioblastoma, histone deacetyase inhibitors, PARP-1 inhibitors

## Abstract

Glioblastoma, a fatal disease in both adult and pediatric patients, currently has limited treatment options that offer no more than temporary relief. Our experiments with adult and pediatric glioblastoma cell lines showed that radiation induces a dose-dependent increase in the level of MutT homolog 1 (MTH1) - an enzyme that hydrolyzes oxidized purine nucleoside triphosphates. Similarly, the combination of vorinostat, which is a histone deacetylase inhibitor, and ABT-888, which is a PARP-1 inhibitor, enhanced clonogenic death and increased the MTH1 level, relative to each treatment alone. This result suggests that the MTH1 level is directly related to the damage that is inflicted upon the cells, and its activity protects them against anti-neoplastic therapy. Indeed, the MTH1 inhibitor TH588 and MTH1 siRNA increased glioblastoma's response to both radiation and the combination of vorinostat and ABT-888. TH588 also inhibited glioblastoma's capacity for migration and invasion. In normal fibroblasts, low radiation doses and the combination of vorinostat and ABT-888 decreased the level of the enzyme. TH588 did not alter the fibroblasts’ response to radiation and only mildly affected their response to the combination of vorinostat and ABT-888.

In summary, the inhibition of MTH1 is required to better realize the therapeutic potential of anti-neoplastic treatments in glioblastoma.

## INTRODUCTION

Glioblastoma is the most severe type of human brain tumor in both adults and children, with a median survival of 14.6 months for adults [1] and 13–18 months for pediatric patients [2, 3]. The current standard treatment for adult glioblastoma consists of the sequential introduction of three modalities: a maximal-safe surgical resection, followed by radiotherapy in combination with temozolomide [1]. In contrast to adults, a significant percentage of pediatric patients’ glioblastoma are located in the midline region of the brain and either, like diffuse intrinsic pontine glioma (DIPG), are completely inaccessible for surgical resection or, like the diffuse thalamic midline gliomas, are not favorably recommended for it. In pediatric patients, the addition of temozolomide failed to show any survival benefit compared to radiation therapy alone [4, 5].

The lack of effective treatments for glioblastoma patients has led to a continuing search for new therapies. In particular, a great effort has been directed toward understanding the molecular characteristics of glioblastoma, which could provide clues to the disease's etiology and point to new molecular targets for drug development. Thus, genome analysis of tumors from primarily pediatric glioblastomas [6–8] as well as adult glioblastomas [9] revealed the existence of somatic heterozygous histone mutations that are mutually exclusive (i.e., different subtypes of glioblastoma that originate in different regions of the brain are associated with different specific mutations). Therefore, the H3.3K27M and H3.1K27M mutations are associated with DIPG and other midline gliomas, while the H3.3G34R/V mutation has been found in hemispheric gliomas [10–12]. The K27M mutation leads to increased activity of both chromatin domains that suppress differentiation and of those that support proliferation. Notably, both types are essential for tumor survival [13–15].

In addition to molecular studies, the screening of chemical libraries and anti-cancer drugs aimed at the discovery of potentially effective treatments has also been conducted. In one such study, Funato et al. [16] revealed that the inhibitor of menin - a member of the trithorax family histone methyltransferase complex - inhibited the growth of human DIPG cells both *in vitro* and, in the xeongraft model, *in vivo*. Grasso et al. [17] demonstrated the inhibitory effect of panobinostat, which is the histone deacetylase inhibitor (HDACi), on the growth of DIPG human cell lines both *in vitro* and, in the tumor xenograft model, *in vivo*. In addition, van Vuurden et al. showed that the PARP-1 inhibitor (PARPi) olaparib sensitized pediatric high grade glioma to ionizing radiation [18]. The findings that histone deacetylase inhibitors sensitize breast, ovarian, prostate, and myeloid leukemia cancer cells to treatment with PARP-1 inhibitors, regardless of their innate capacity for repair of dsDNA breaks [19–22], are relevant to these studies. Our work, which focused on breast cancer cells that are inherently resistant to PARP-1 inhibitors, showed that vorinostat sensitized them to the inhibition of PARP-1 [20].

Similar to ionizing radiation and chemotherapy, HDACis lead to increased levels of reactive oxygen species (ROS), which participate in mediating their toxic effect [23–25]. Increased levels of ROS lead to increased levels of 8-Oxo-dGTP in the cellular nucleotide pool, which can lead to increased mutations and/or futile base excision repair and cell death when they enter the DNA. Therefore, any cellular machinery that counteracts the effect of 8-oxo-dGTP is likely to diminish the effect of the ROS production by anti-neoplastic treatments. Indeed, the survival of cancer cells that maintain a high metabolic rate relative to normal cells are dependent on the activity of the MTH1 enzyme, which prevents the incorporation of 8-oxo-dGTP into the DNA by hydrolyzing it into 8-oxo-dGMP and pyrophosphate. Thus, it has been suggested that MTH1, which supports accelerated proliferation of cancer cells, may also block anti-cancer therapies [26–29]. In addition to its effect on cancer cell survival and proliferation, recent studies have shown that abrogation of MTH1 expression in RAS-transformed lung cells decreased their capacity for migration [30].

In the present study, we show that, similar to what has been found with other human cancers [19–22, 31], the combination of HDACi and PARPi (vorinostat, a pan-HDACi, and ABT-888) increased clonogenic death in both pediatric and adult glioblastoma cell lines. Importantly, the combined treatment led to increased levels of ROS and of MTH1, relative to each treatment alone and to untreated cells. Inhibition of MTH1 activity in cells treated with both vorinostat and ABT-888 led to a further decrease in cell survival. These experiments also revealed that inhibition of MTH1 activity in-itself could increase the sensitivity of glioblastoma to treatment with PARP-1. Similarly, as noted by others [32, 33], ionizing radiation led to an increased level of MTH1. Again, the inhibition of MTH1 activity in irradiated cells enhanced their clonogenic death, relative to cells treated with radiation alone. The inhibition of MTH1 activity in normal fibroblasts did not alter their response to ionizing irradiation and had a milder effect on their response to the combination of vorinostat and ABT-888 than that observed in the glioblastoma cells. In addition, the inhibition of MTH1 activity in glioblastoma interferes with its capacity for cell migration and invasion

Our results suggest that, in glioblastoma, MTH1 activity serves as a defense mechanism against the cytotoxic effect of anti-neoplastic treatments. The activity of MTH1 should therefore be inhibited to better exploit the potential of the applied therapy.

## RESULTS

### Combined treatment of vorinostat and ABT-888 leads to enhanced clonogenic death in glioblastoma cell lines

**Figure 1:**
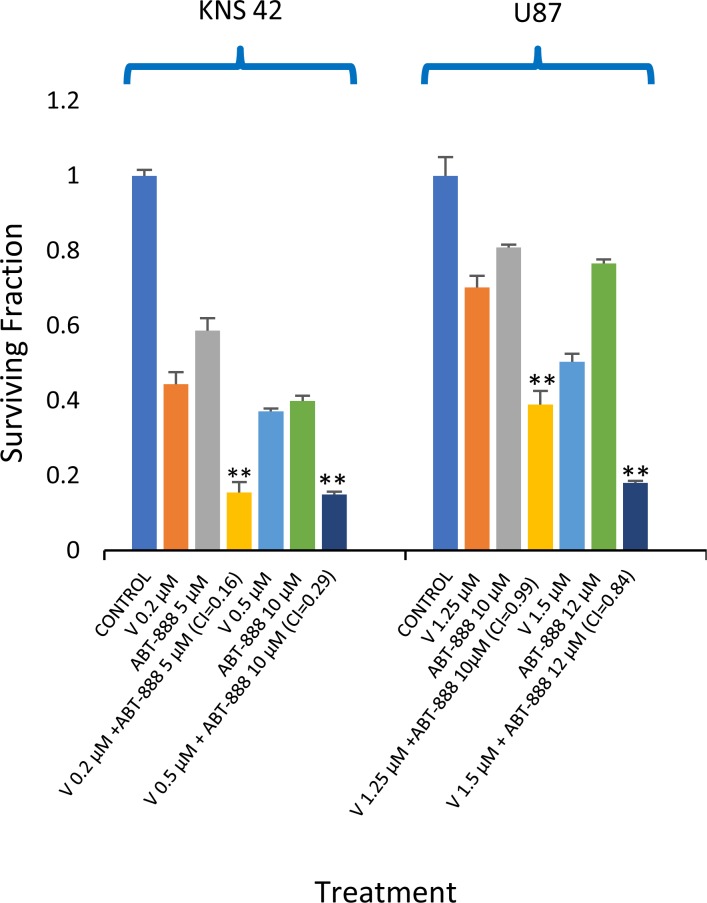
Vorinostat increases the sensitivity of glioblastoma to ABT-888: Colony survival assay and derivation of CI values was conducted as described in methods Values are mean of surviving fraction ± S.E.M of triplicates. Stars represent significant differences between the surviving fraction of the combination treatment and each one of its components. ^**^p<0.005.

**Figure 2:**
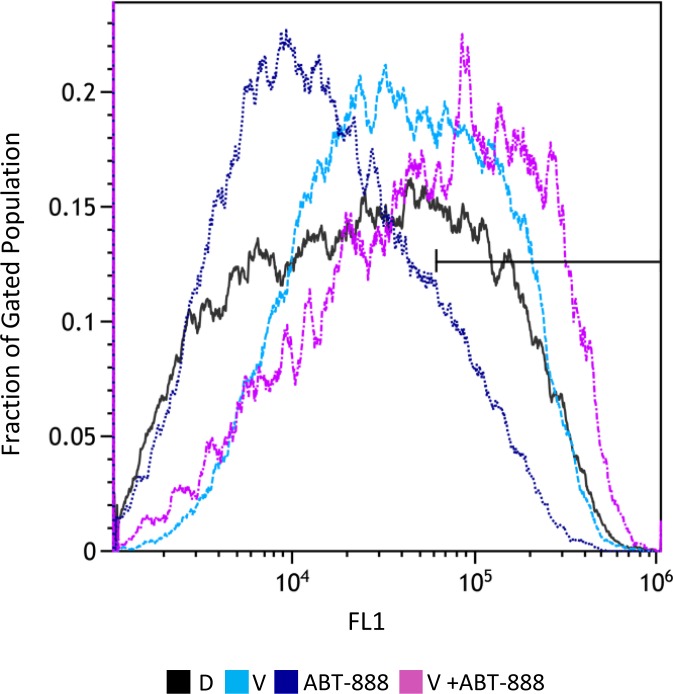
Combined treatment of vorinostat and ABT-888 increases cellular level of ROS: Cells were treated with 0.5 μM vorinostat (V), 10 μM ABT-888 or both Control untreated cells received DMSO (D). Seventy-two hours later, the cells were stained with CM-H_2_DCFDA and their ROS level was evaluated by flow-cytometry measurements of their fluorescent intensity as described in Methods. The bar indicates that the combination of vorinostat and ABT-888 increases the fraction of cells with the highest fluorescent intensity (X-MED ~6^*^10^4^) relative to cells treated with vorinostat or to controls. Treatment with ABT-888 leads to decreased level of ROS in the cells relative to control untreated cells. X-Med values for control, ABT-888 and vorinostat were ~2.6^*^10^4^, 1.3^*^10^4^, and 3.9^*^10^4^, respectively.

We have previously shown that vorinostat can sensitize human breast cancer cells to ABT-888, regardless of their BRCA status [20]. Here, we show that the combined treatment of vorinostat and ABT-888 leads to increased clonogenic death of both pediatric and adult glioblastoma cell lines. The CI values point to a synergistic interaction of the drugs in KNS42. In U87, the interaction was additive at the lower concentration range and synergistic at the higher concentration range. (Figure 1). Increased levels of ROS are known to mediate the toxic effect of several anti-neoplastic treatments (23-25). However, under our experimental conditions and similar to what has been reported by others [34, 35], vorinostat and PARP-1 inhibitors had an opposite effect on the cellular level of ROS. While vorinostat increased the level of ROS, ABT-888 led to its decrease. Nonetheless, the combined administration of vorinostat and ABT-888 increased the fraction of cells with the highest ROS level relative to that obtained in controls, or in cells treated with vorinostat or PARP-1 inhibitor alone (Figure 2).

### The combination of vorinostat and ABT-888 increases the level of MTH1 in glioblastoma cell lines

**Figure 3:**
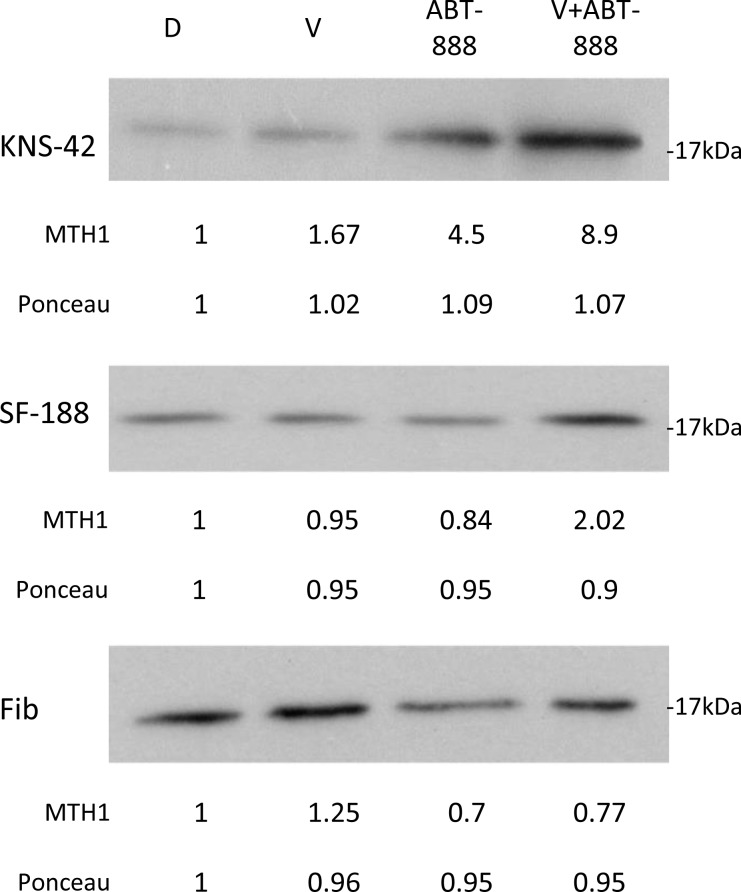
Combined treatment of vorinostat and ABT-888 increases cellular level of MTH1 in glioblastoma cells: Cells were treated with 0.5 μM vorinostat (V), 10 μM ABT-888 or both Control untreated cells received DMSO (D). Fourty-eight hours later the cells were processed for Western blot analysis as described in Methods. Numbers at the bottom of the autoradiograms represent amounts of loaded proteins (ponceau) relative to control untreated cells and treatment-induced changes in the level of MTH1. Fib. – Fibroblasts.

Cancer tissues are dependent on MTH1 activity for their survival due to their high levels of ROS [36, 37]. In addition, it has been demonstrated that ionizing radiation and kainate administration, which induce oxidative stress and elevate the level of ROS, lead to an increased cellular level of the MTH1 protein [23, 38]. As shown in Figure 3, we determined the effect of vorinostat, ABT-888, and their combination on the level of MTH1 in KNS42, SF188, and normal fibroblasts. In KNS42 and SF188, the combined treatment of vorinostat and ABT-888 led to an increased level of MTH1, relative to each one of the drugs. In contrast to glioblastoma, normal fibroblasts responded to the combined treatment by reducing the level of the enzyme. The increased level of MTH1 in treated cancer cells could potentially counteract the anti-neoplastic effect of the applied treatment. We therefore tested the effect of MTH1 inhibitors on the survival of glioblastoma cell lines and normal fibroblasts.

### The effect of MTH1 inhibitors on the clonogenic survival of glioblastoma and normal fibroblasts

**Figure 4:**
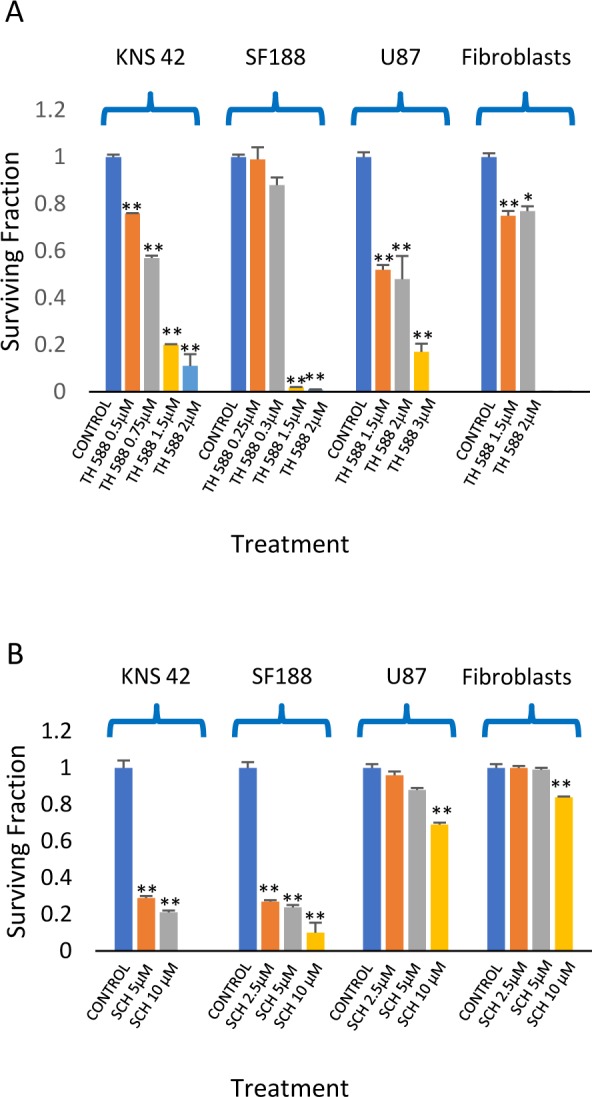
The effect of MTH1 inhibitors on the clonogenic survival of glioblastoma and fibroblasts: Colony survival assay was conducted as described in methods Values are mean of surviving fraction ± S.E.M of triplicates. **(A)** TH588. **(B)** SCH5134. The stars represent a significant difference between the surviving fractions of the treated cells and controls. ^*^p<0.05, ^**^p<0.005.

**Figure 5:**
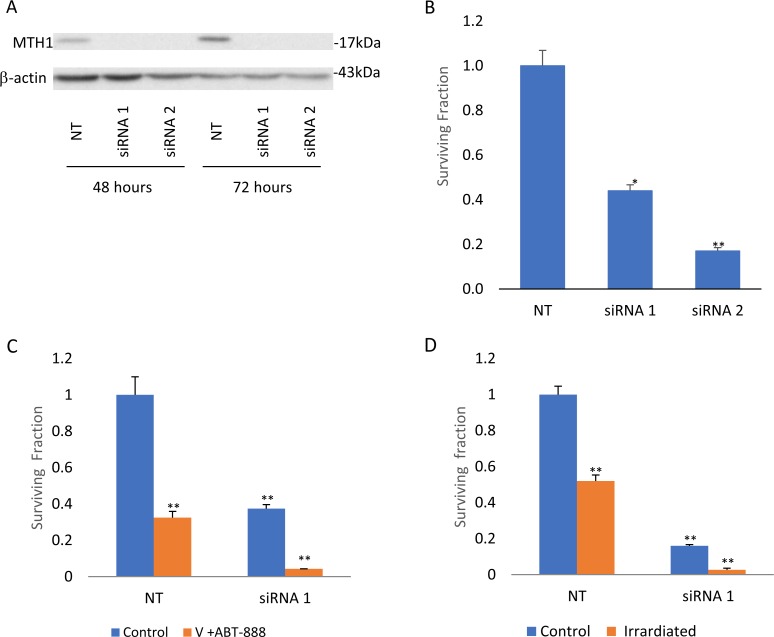
Abrogation of MTH1 expression decreases survival and increases sensitivity to combined treatment of vorinostat and ABT-888 and to ionizing irradiation: KNS-42 cells were transfected with MTH1 and control non-targeting (NT) siRMAs and processed for Western blot analysis or cell survival assays as described in methods **(A)** Transfection with two different MTH1 siRNAs leads to decreased MTH1 expression by more than 80%. Inhibition of MTH1 expression led to decreased cell survival **(B)** and to increased sensitivity to combined treatment of vorinostat and ABT-888 **(C)** or to ionizing radiation **(D)**. Values are mean of surviving fraction ± S.E.M of triplicates. ^*^p<0.05, ^**^p<0.005.

We employed two different MTH1 inhibitors: TH588 and SCH51344. TH588 was developed by Gad et al. while SCH51344 was initially discovered through the screening of chemical libraries for inhibitors of RAS-transformed cells, [39, 40], and later studies demonstrated that MTH1 is its cellular target [41]. As noted in Figure 4, KNS42 and SF188 were sensitive to both inhibitors. SF188 cells, which carry a focal deletion of neurofibromatosis 1 at 17q11.2 [42], showed the highest sensitivity to TH588. This mutation leads to activation of RAS [43] and, like RAS-mutated cancer cells, may be extremely sensitive to MTH1 inhibition due to their high metabolic and ROS production rate [29]. The U87s were extremely sensitive to TH588 and less sensitive to SCH1344. Similarly, abrogation of MTH1 expression by two different MTH1 siRNA led to decreased survival of glioblastoma cells (Figure 5A, 5B). Normal fibroblasts responded poorly to both TH588 and SCH1344 (Figure 4). A similar effect of TH588 on the survival of glioblastoma cells has recently been demonstrated by Pudelko et al. [44].

### The effect of the MTH1 inhibitor on the cellular response to combined treatment of vorinostat and ABT-888

**Figure 6:**
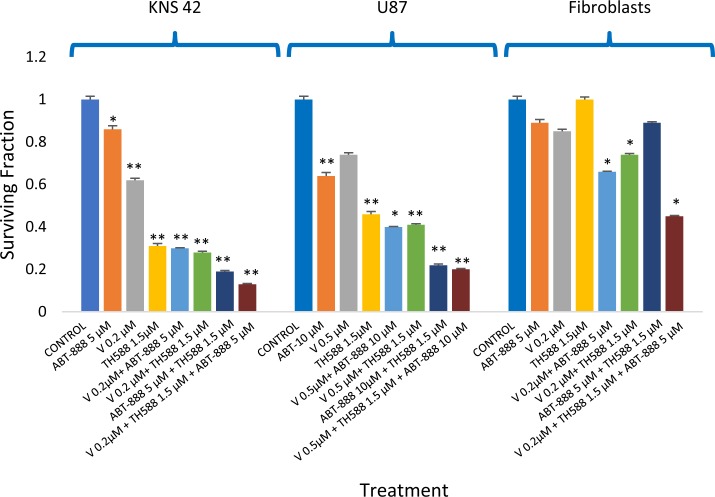
Inhibition of MTH1 increases the response of glioblastoma to the combination of vorinostat and ABT-888: Colony survival assay was conducted as described in methods Values are mean of surviving fraction ± S.E.M of triplicates. The stars represent significant differences between treatments with single agents and control, between combinations of two agents and each one of their components and between a combination of three agents and the combinations of TH588 and vorinostat, TH588 and ABT-888 and vorinostat and ABT-888. For U87 significance was similar to that observed in KNS42 except for the minor insignificant difference in survival between the triplicate and the combination of TH588 and ABT-888. ^*^p<0.05, ^**^p<0.005.

The fact that the combined treatment of vorinostat and ABT-888 leads to an increased level of MTH1, suggests that the activity of this enzyme may hamper the therapeutic potential of these drugs. The results shown in Figure 6 demonstrate that inhibition of MTH1 activity in cells treated with both vorinostat and ABT-888 does indeed increase their clonogenic death. In KNS42 and U87, the addition of TH588 to the combination of vorinostat and ABT-888 reduced their clonogenic survival by half relative to the combination treatment and to TH588 alone. Similarly, MTH1 siRNA reduced clonogenic survival of KNS42 and increased their sensitivity to the combined treatment of vorinostat and ABT-888 (Figure 5C). In fibroblasts, the addition of TH588 to the combination of vorinostat and ABT-888 resulted in a milder ~30% reduction in their clonogenic survival relative to the combination of vorinostat and ABT-888 (Figure 6).

### The MTH1 inhibitor increased sensitivity to the PARP-1 inhibitor in glioblastoma cell lines

**Figure 7:**
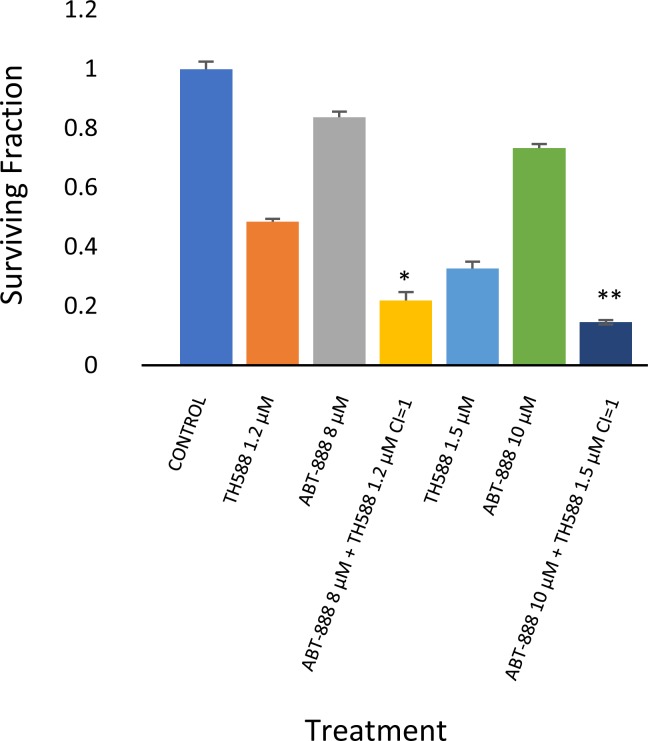
MTH1 inhibitor increased sensitivity to PARP-1 inhibitor in KNS42: Colony survival assay and derivation of CI values was conducted as described in methods Values are mean surviving fraction ± S.E.M of triplicates. Stars represent significant differences between a combination treatment and each one of its components. ^*^p< 0.05, ^**^p<0.005.

The experiment shown in Figure 6 suggests that TH588 can increase the response of glioblastoma to PARP-1. Indeed, as noted in Figure 7, the inhibition of MTH1 activity increased the response of KNS42 to the PARP-1 inhibitor. The CI numbers indicate an additive interaction between the two drugs.

### Inhibition of MTH1 activity increases DNA damage caused by the combined treatment of vorinostat and ABT-888

**Figure 8:**
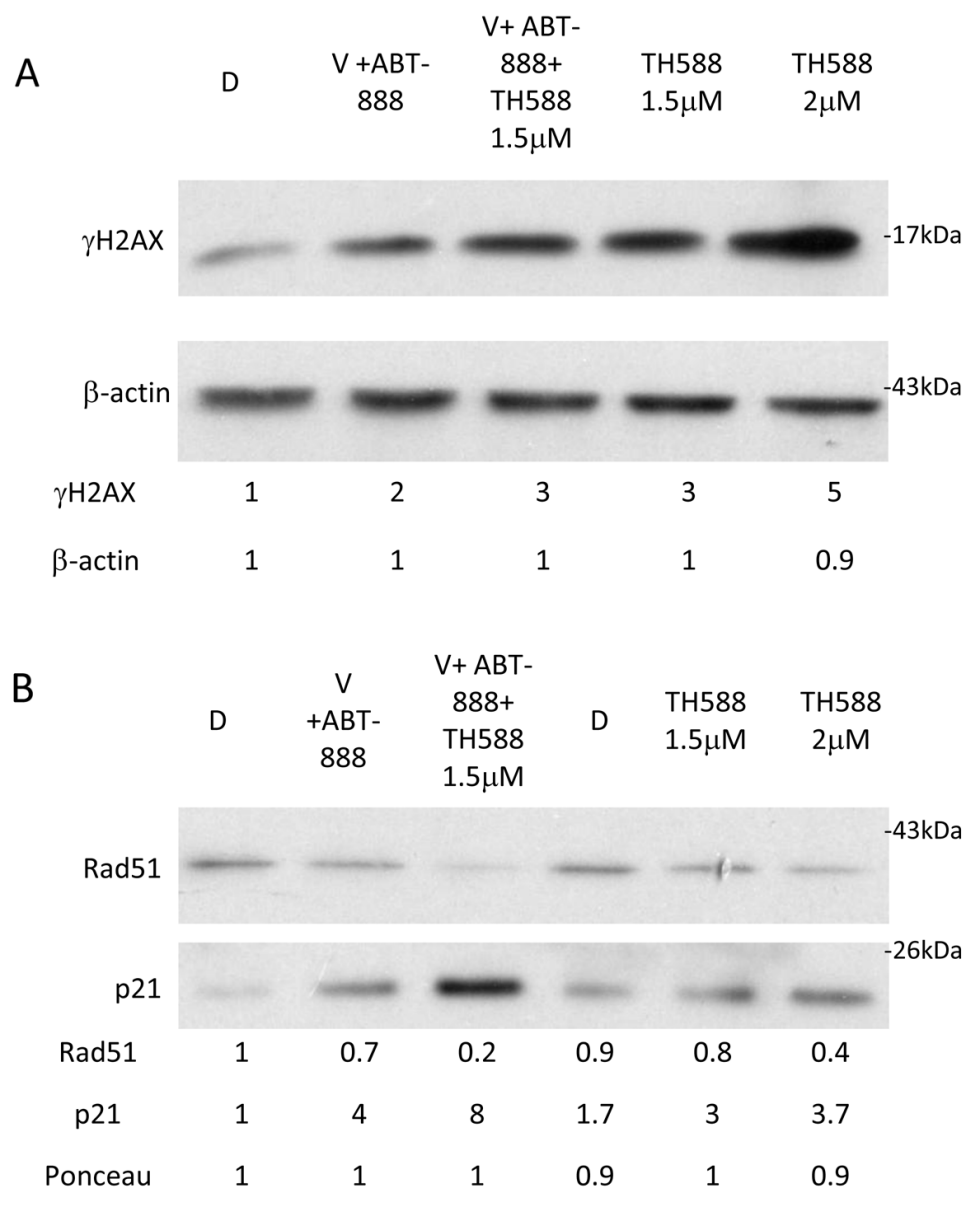
The effect of MTH1 activity on the level of γ-H2AX, RAD51 and p21 in cells treated with both vorinsotat and ABT-888: Cells were treated with the specified drugs for 48 hours and processed for Western blot analysis as described in methods V (0.5 μM vorinostat), ABT-888 (10 μM), TH588 (1.5 μM in combination with V and ABT-888), **(A)** Numbers at the upper row represent treatment induced change in the level of γ-H2AX relative to control and at the lower raw treatment induced change in the level of β-actin (loading control). **(B)** Numbers at the two upper rows represent treatment induced changes in the level of RAD51 and p21 and at the lower row the amounts of loaded proteins (ponceau) relative to control untreated cells. The p21 row is composed of two different experiments: 1. D, V+ABT-888, V+ABT-888+TH588 and 2. D, TH5881.5uM and TH5882uM

**Figure 9:**
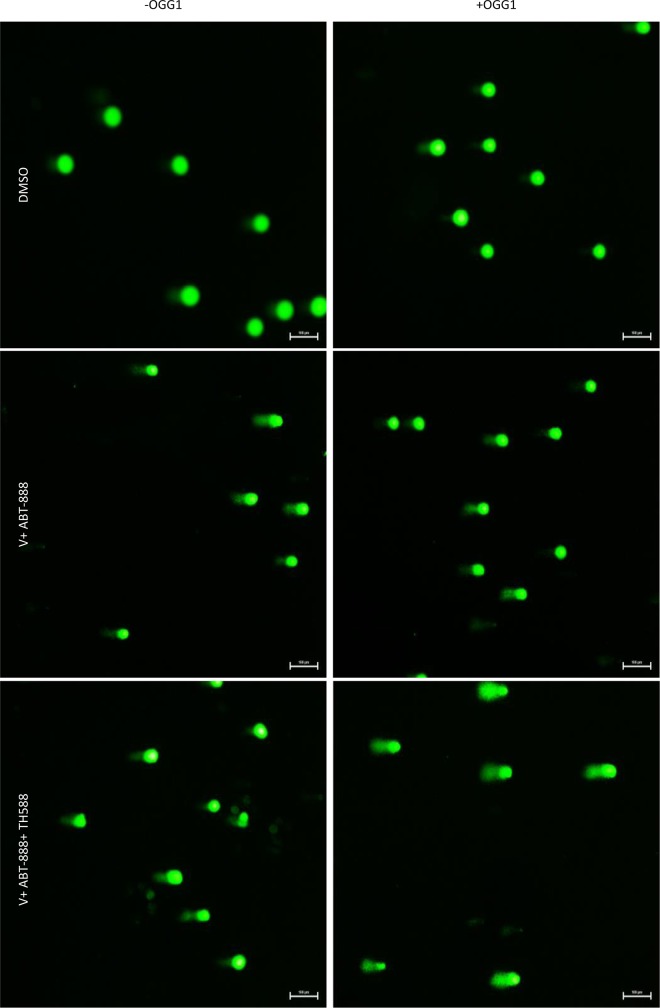
The effect of MTH1 on DNA damage in cells treated with both vorinostat and ABT-888: Cells were incubated with the specified drugs and the effect of hOGG1, on the results obtained with the comet assay were tested as described in methods

**Figure 10:**
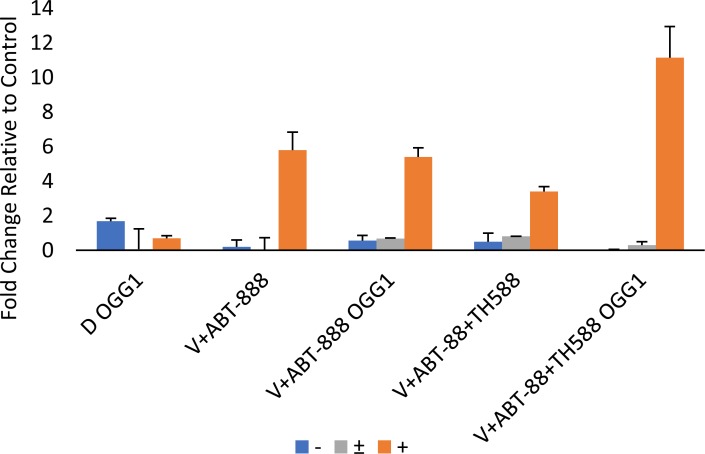
Quantification of the results obtained in the comet assay: Quantification was performed as described in methods DMSO (D). V+ABT-888 (cells treated with vorinostat and ABT-888), V+ABT-888+TH588 (cells treated with vorinostat, ABT-888, and TH588). OGG1, Slides are treated with hOGG1(+) or with its vehicle OGG1 (-) before electrophoresis. Numbers are average ± SEM of fold change relative to control from two independent experiments (100 comets were counted in each sample). Cells treated with V+ABT-888+TH588 + OGG1 displayed the highest fraction of cells with long comets.

Increased phosphorylation of γ-H2AX serves as an indicator for DNA damage [45]. As noted in Figure 8, TH588 led to a dose-dependent increase of γ-H2AX. The level of γ-H2AX in cells that were treated with the combination of the three drugs was higher than that detected following treatment with the combination of vorinsotat and ABT888 but similar to that obtained with 1.5 μM TH588 alone. Importantly, while the combined treatment of vorinostat and ABT-888 led to only a moderate reduction in the cellular level of RAD51 addition of TH588 to this treatment led to a drastic reduction in the cellular level of the DNA repair protein. By itself TH5888 also led to a dose-dependent decrease of RAD51, albeit to a more moderate than that obtained by the three inhibitors combined. These results are in line with those obtained with the alkaline comet assay that employ hOGG1 in order to determine the effect of the various treatments on the incorporation of 8-Oxo-dG into the DNA (Figures 9, 10). hOGG1 is a base excision repair protein that excises 8-Oxo-G when 8-Oxo-dG is incorporated opposite cytosine [46]. Its activity in the context of the comet assay increases the level of DNA breaks and therefore the fraction of cells with comets and the amount of DNA in the comets. We tested the effect of hOGG1 on the appearance of comets in cells treated with both vorinostat and ABT-888 and in cells treated with the combination of vorinsotat, ABT-888 and TH588. While all treatment led to increased appearance of long comet relative to controls, there was no difference in the appearance of comets between vorinoatat and ABT-888 or vorinostat, ABT-888 and TH588 treated cells. Also, hOGG1 did not increase the appearance of comets in cells that were treated with vorinostat and ABT-888. However, a dramatic effect of hOGG1 on the appearance of comets was observed in cells that were treated with vorinostat, ABT-888 and TH588. Here an 11 fold increase in the appearance of comets relative to control was observed (Figure 10).

Interestingly, although the combination of vorinostat and ABT-888 increased the level of ROS in the cells, under our experimental conditions it did not lead to a significant increase in the incorporation of 8-Oxo-dG into the DNA, most probably due to elevated level of MTH1. However, inhibition of MTH1 activity in these cells resulted in a significant increase in the incorporation of 8-Oxo-dG into their DNA.

### The effect of MTH1 activity on the sensitivity to ionizing radiation

**Figure 11:**
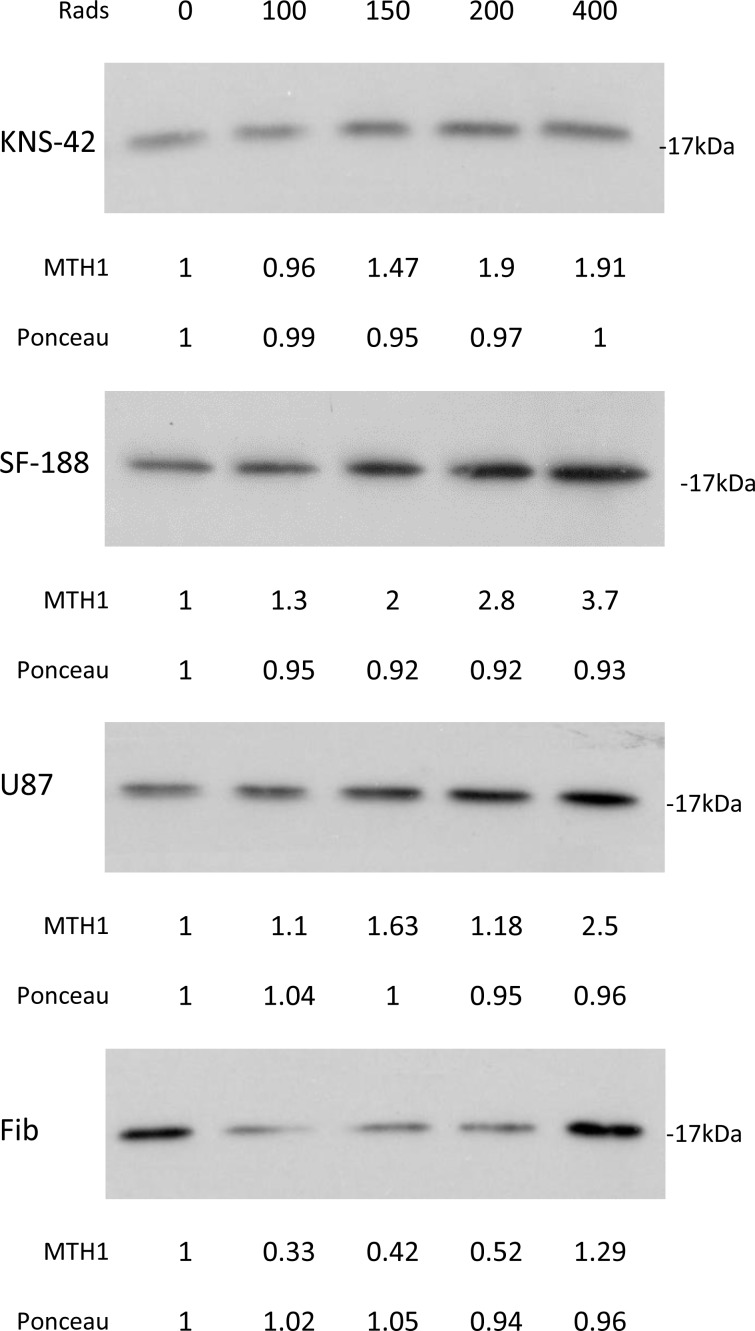
The effect of ionizing radiation on cellular level of MTH1 in glioblastoma and normal fibroblasts: Cells were irradiated and 48 hours later were processed for Western blot analysis as described in methods Numbers at the bottom of the autoradiograms represent treatment-induced changes in the level of MTH1 and amounts of loaded proteins (ponceau) relative to control untreated cells. The experiment was reproduced once with similar results.

**Figure 12:**
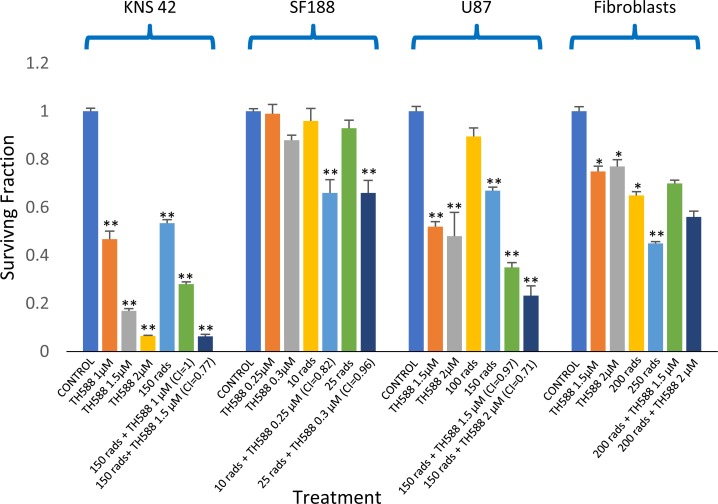
The effect MTH1 inhibitor – TH588 – on the sensitivity to ionizing irradiation of glioblastoma and fibroblasts: Cells were plated for colony survival assay and derivation of CI values were described in methods Values are mean surviving fraction ± S.E.M of triplicates. Stars represent significant differences between a single agent and control and between a combination treatment and each one of its components. Fib. – Fibroblasts. ^*^p<0.05, ^**^p<0.005.

As shown in Figure 11, radiation led to a dose-dependent increase in the cellular level of MTH1 in the glioblastoma cell lines. Interestingly, in normal fibroblasts, treatment with 100, 150, and 200 rads decreased the level of MTH1, while 400 rads increased the cellular level of the enzyme. The addition of an MTH1 inhibitor to cancer cells, which had been irradiated with clinically relevant radiation doses [1, 4], led to increased clonogenic death, relative to each agent alone (Figure 12). Because SF188 cells are extremely sensitive to the inhibition of MTH1 (Figure 3), we employed low concentrations of TH588 (0.25 and 0.3 μM) in combination with extremely low doses of radiation (10 and 25 rads). Nonetheless, these combinations led to increased clonogenic death, which suggests that the inhibition of MTH1 may allow for the inclusion of a low radiation doses in clinical protocols for the treatment of pediatric glioblastoma with hyper-activated RAS. In KNS42 and U87 the nature of the interaction between the MTH1 inhibitor and the radiation was either additive at the lower concentration range or slightly synergistic at the higher concentration range. Here too, the results obtained with MTH1 siRNA were similar to those obtained with TH588. Abrogation of MTH1 expression increased the sensitivity of the cells to ionized irradiation (Figure 5D). The addition of TH588 to irradiated fibroblasts did not lead to a statistically significant increase in their clonogenic death. This result is in line with the fact that, under our experimental conditions, the radiation itself leads to a decreased level of the enzyme in normal fibroblasts (Figure 11).

### The effect of MTH1 activity on cell migration and invasion

**Figure 13:**
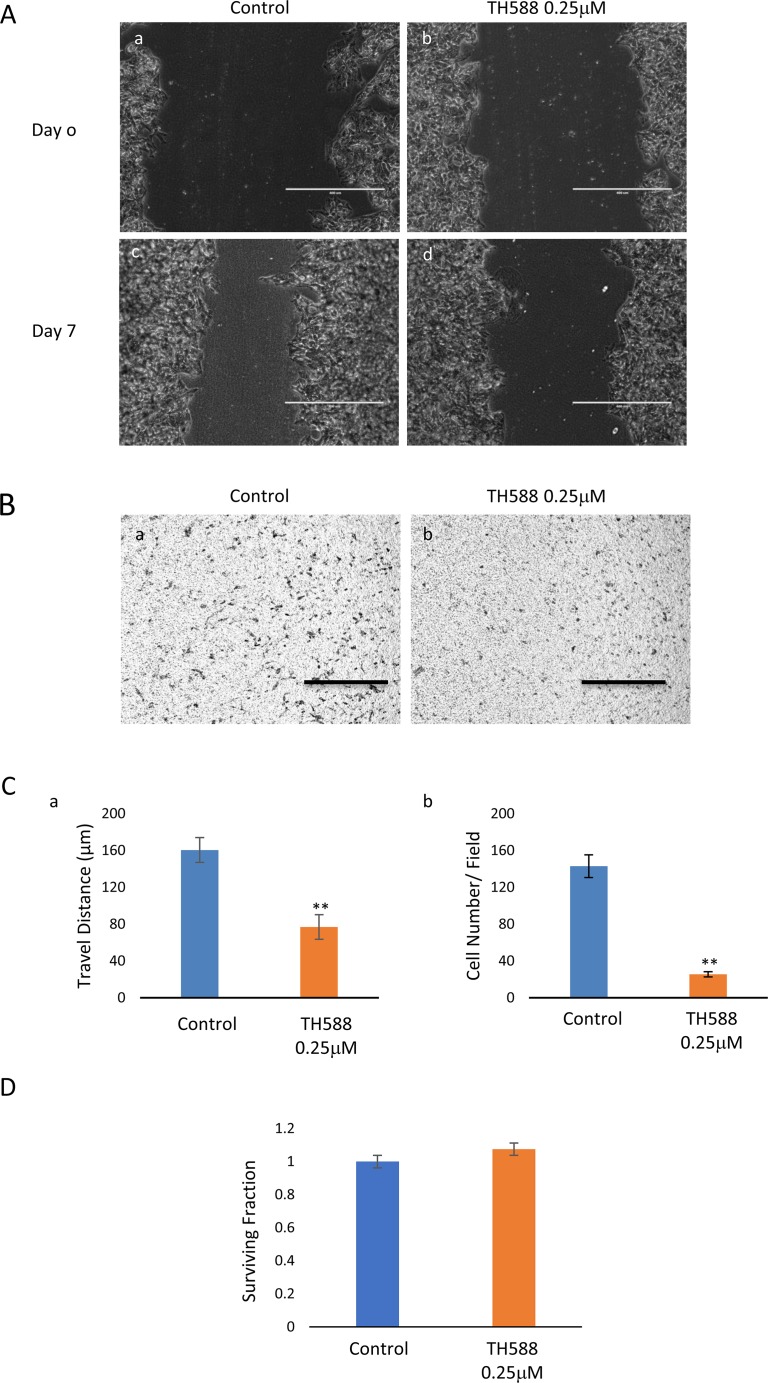
MTH1 inhibitor interferes with cell migration and invasion: **(A)** The inhibitory effect of TH588 on KNS42 migration was manifested in the wound-healing assay as a difference in the width of the scratch between control and TH588 treated cells. Control untreated cells at time 0 (a) and four days later (b). TH588 treated cells at time 0 (c) and four days later (d). Bar, 400 μM. **(B)** TH588 inhibits the capacity of KNS24 for invasion: Control (a,c) and TH588 treated cells (b,d). Bar, 100 μM. **(C)** The extent of TH588 inhibition of KNS42 migration (a) and invasion (b) are presented in the histograms. Differences between control and experimental was specific p<0.005. Numbers are mean ± S.E.M of the travelled distance in μM measured at 10 different fields (a) and of cells at the bottom side of the insert at the end of the experiment counted at 5 different fields (b). **(D)** TH588 at 0.25 μM does not affect the clonogenic survival of KNS42.

As noted in Figure 13, low concentrations of TH588, which do not affect the clonogenic survival of the cells, interfered with the KNS42 cells’ capacity for cell migration and invasion.

## DISCUSSION

Cancer cells maintain a higher metabolic rate than normal cells and therefore produce a higher level of ROS. High levels of ROS result in the harmful oxidation of cellular components, such as proteins, lipids, and nucleotides [26, 29, 39]. Among the nucleotides in the soluble pool, dGTP and GTP are the most susceptible nucleotides to oxidation [26]. Gad et al. developed MTH1 inhibitors that inhibited the purified soluble enzyme as well as the intracellular enzyme *in situ*. The effect of TH588 on the accumulation of 8-Oxo-dG, on clonogenic survival and on tumor development was similar to that obtained following abrogation of MTH1 expression with specific RNAis [39, 47]. Notably, in human embryonic pulmonary fibroblasts, long-term abrogation of MTH1 expression by RNAi led to increased expression of 8-Oxoguanine (8-Oxo-G) DNA glycosylase 1 (OGG1) - an enzyme that could compensate for the loss of the MTH1 by removing 8-OxoG, which are found opposite to cytosine [48]. Thus, abrogation of MTH1 expression may not always recapitulate the toxic effect of MTH1 inhibitors [47, 49].

Our studies evaluated the effect of MTH1 inhibitors and MTH1 siRNA on the sensitivity of glioblastoma to treatments with vorinostat and ABT-888 as well as to ionizing irradiation. The results showed that exposing glioblastoma to the combined treatment of vorinostat and ABT-888 led to increased clonogenic death. Interestingly, the combined treatment also led to an increase in the fraction of cells with the highest level of ROS and in the cellular level of MTH1, relative to each treatment alone. However, the increased level of MTH1 protein could not be triggered by increased ROS alone as a moderate increase in the level of MTH1 was observed in ABT-888 treated cells that showed a reduction in cellular level of ROS. Nonetheless, the increased level of MTH1 following the co-administration of vorinostat and ABT-888 prompted us to examine the possibility that the activity of MTH1 decreases the efficiency of this combined treatment. We first tested the effect of the MTH1 inhibitors TH588 and SCH51344 on the clonogenic survival of KNS42, SF188, U87, and normal fibroblasts and found that the glioblastoma were sensitive to the inhibition of MTH1 activity, while normal fibroblasts were barely affected. As expected, the addition of TH588 to cancer cells treated with both vorinostat and ABT-888 further reduced their clonogenic survival by 50%. In addition, the inhibition of MTH1 increased the response of cancer cells to the PARP-1 inhibitor. It is possible that inhibition of the MTH1, which over time may result in the formation of ssDNA breaks, triggers the binding of PARP-1 as a prelude to the repair of those breaks. The inhibition of PARP-1 will inhibit the repair of ssDNA breaks and increase cell death. To the best of our knowledge, this mode of interaction between the inhibitors of PARP-1 and MTH1 has not been reported to date.

Our experiments showed that inhibition of MTH1 activity is associated with increased level of the DNA damage marker, γ-H2AX, and at the same time to a decreased level of the DNA repair protein, RAD51 and also increases the level of cyclin-dependent kinase inhibitor 1 (p21) which leads to the arrest of cell cycle progression. In addition, under our experimental conditions, the comet experiments indicate that while the combined treatment of vorinostat and ABT-888 induced DNA damage it did not lead to a detectable increase in 8-Oxo-dG in the DNA. In contrast, inclusion of TH588 in the combined treatment led to a dramatic increase in the DNA level of 8-Oxo-dG. This observation suggests that indeed increased level of MTH1 following administration of vorinostat and ABT-888 diminish the harmful effect of the applied treatment.

Recently, it has been demonstrated that ionizing radiation induced an increase in the level of both MTH1 mRNA and protein [32, 33]. We also found that irradiation of cancer cells with 100, 150, 200, and 400 rads led to an increased level of the MTH1 protein in cancer cells. Curiously, radiation doses of 100, 150, and 200 rads led to a decreased level of MTH1 in normal fibroblasts, while 400 rads led to an increased level of the protein in these cells. The addition of MTH1 inhibitors to cancer cells following irradiation with clinically relevant doses (100–200 rads) led to increased clonogenic death of cancer cells, relative to radiation treatment alone. The interaction between the MTH1 inhibitor and radiation was either additive under the milder conditions or mildly synergistic under harsher conditions (i.e., higher concentrations of the MTH1 inhibitor and higher radiation doses). This result is of vital importance to pediatric glioblastoma patients, whose main treatment-option is ionizing radiation. An increased response to ionizing radiation in TH588-treated neuroendocrine tumor cell lines has recently been reported by Prada et al. [50]. In contrast to the glioblastoma cell lines, the MTH1 inhibitor failed to affect the clonogenic survival of irradiated fibroblasts.

Recent studies with breast cancer-expressing oncogenic RAS showed that abrogation of MTH1 expression led to both decreased expression of E-cadherin and suppression of the mesenchymal phenotype, which are both features that are associated with cancer cells’ capacity for migration and invasion [29, 30]. Our results showed that low concentrations of TH588, which by themselves do not affect clonogenic survival, inhibited both cell migration and invasion.

It has been reported that that the activity of MTH1 protects the brain against oxidative damage [38]. Our studies demonstrated that inhibitors of MTH1 decrease clonogenic survival of glioblastoma cells and increase their response to therapy. This is of particular relevance to pediatric glioblastoma, where the addition of temozolomide to ionizing radiation does not result in delayed tumor progression. By contrast, in adult glioblastoma, the standard treatment is combined temozolomide and radiation. Therefore, localized inactivation of MTH1 activity, such as convection-enhanced delivery [17], may have to be developed. In addition, understanding the molecular mechanisms that underlie the increased level of MTH1 in treated glioblastoma may lead to the finding of cellular targets for the development of drugs that will specifically inhibit the expression of MTH1 in these tumors.

## MATERIALS AND METHODS

### Cell lines

Short, tandem, repeat profiling for authentication of U87, SF-188 and KNS42 was performed by the Genomic Center Core Facility (Technion, Haifa). The human glioblastoma cell line U87 from the American Type Culture Collection (Manassas, VA) was authenticated in 2014, and its frozen aliquots are being resuscitated and used for eight weeks. The SF188 cell line was kindly provided in 2014 by Dr. Daphne Haas-Kogan (UCSF, San Francisco, CA). This cell line was authenticated in 2017, and frozen aliquots were prepared and are being resuscitated and used for eight weeks. The pediatric KNS-42 cell line expressing H3.3G34V, which was authenticated by short, tandem, repeat profiling, was purchased in 2012 from the Japanese Collection of Research Bioresources Cell Bank(Osaka, Japan). Following a short propagation period, the cells were frozen, and aliquots were resuscitated and used for eight weeks. The cell line was re-authenticated again recently as mentioned above. Fibroblasts were obtained from dermal human fibroblasts, per protocol #7044 (approved by the Institutional Review Board at Sheba Medical Center).

### Growth conditions

U87 cells were grown in high-glucose Dulbecco's modified Eagle's medium (DMEM), KNS-42 cells were grown in Eagle's minimal essential medium, and SF188 cells were grown in high-glucose DMEM. The media were supplemented with fetal bovine serum (FBS) (5% for KNS-42 and 10% for SF188 and U87), penicillin, and streptomycin (Biological Industries, Kibbutz Beit-Haemek, Israel). Fibroblasts were grown in high-glucose DMEM that was supplemented with 20% FBS, penicillin-streptomycin, 1% non-essential amino acids, and 0.2% β-mercaptoethanol (Invitrogen, Life Technologies, Grand Island, NY). For the Western blot analysis of cellular proteins, cells were plated at a density of 4.10^3^ per cm^2^, and treatment was initiated 48 hours post-plating. Drugs were added from stock solutions in dimethyl sulfoxide (DMSO) (Sigma, St. Louis MO), and the controls received the vehicle. The concentration of DMSO in the growth medium did not exceed 0.08%. Crystallized trypsin was also from Biological Industries.

### Reagents

Vorinostat was obtained from LC laboratories (Boston, MA), and the ABT-888 was from APExBIO (Houston, TX). The MTH1 inhibitors TH588 and SCH51344 were obtained from Sigma (St. Louis Mo) and Tocris Bioscience (Bristol, UK), respectively. hOGG1 FLARE^TM^ assay kit was from Trevigen (Gaithersburg, MD), 5-(and-6)-chloromethyl-2′,7′-dichlorodihydrofluorescein diacetate, acetyl ester (CM-H_2_DCFDA) was obtained from ThermoFisher Scientific (Waltham, MA). SilencerR Select valudated siRNA and non-targeting (NT)-siRNA control were purchased from ambion (Carlsbad, CA) and Lipofectamine RNAiMAX reagent was from Invitrogen (Waltham, MA).

### Radiation

Cells were irradiated in an X-ray irradiator (Polaris sc-500 series II) at a dose-rate of 100 cGy/minute.

### Clonogenic survival

Plating for the colony survival assay, colony counting, and calculation of clonogenic surviving fraction was performed in triplicate, as described previously [51–53]. Treatments (radiation and addition of drugs) were performed 24 hours post-plating. Experimental combination indices (CI) were obtained by employing the computer program CompuSyn, per Chou [53]. CI < 1 indicated synergistic interaction, and C=1 indicated an additive one (Chou TC [53]). All experiments were reproduced at least once. To determine the effect of MTH1 siRNA on cell survival and sensitivity to treatment we employed the following MTH1 siRNAs (Sense sequences: #1. CGACGACAGCUACUGGUUtt #2. CAUCUGGAAUUAACUGGAtt).

For evaluating inhibition of MTH1 expression cells were processed for Western blot analysis as described below. For evaluating the effect of MTH siRNAs on cells’ viability, the cells were seeded in 24 well plates (10,000/well) and transfection with 5 nM MTH1 siRNAs and control non-targeting siRNA was carried out 24 hours later with lipofectamine RNAiMAX reagent according to the manufacturer's instructions. The cells were incubated for five additional days, and then removed by trypsinization mixed with trypan blue for counting of live cells. The effect of siRNA on the sensitivity of the cells to ionizing irradiation or to the combined treatment of vorinostat and ABT-888 was conducted as follows: Cells were seeded in 6 well plates (200,000/well) and transfected with 10 nM siRNA 24 hours later. The cells were then incubated for additional 72 hours. At that time the cells were harvested and seeded in a 6-well dish (500/well). Radiation or addition of drugs was conducted 24 hours post seeding and incubation was continued for 10 more days. Colonies were stained with crystal violet and counted as described above. The experiments were carried out in triplicates and included non-targeting siRNA control.

### Western blot analysis

Preparation of cell lysates and analysis of treatment-induced changes in the protein level were performed, as previously described [52]. Treatment (radiation and addition of drugs) was performed 48 hours post-plating. Protein content was determined with a bicinchoninic acid reagent (Bio-Rad, Hercules, CA), and equal loading was verified by comparing the levels of β-actin or by measuring the absorbance at 520 nm of Ponceau S (Sigma, St-Louis, MO), which was extracted with a phosphate buffer (2.67 mM KCl, 1.47 mM KH_2_PO_4_, 8.1 mM Na_2_HPO_4_, and 1.125 M NaCl) from individual strips of a twin run [51, 52]. Rabbit anti-MTH1 antibodies (NB100-109) and HRP-coupled goat anti-rabbit IgG were obtained from Novous Biologicals (Littlton CO) and Jackson Immunoresearch Laboratories (West Grove, PA), respectively. rabbit anti-γh2AX, anti-RAD51 and anti-p21 were from Cell Signaling Technology (Danvers,, MA) and rabbit-anti β-actin was purchased from abcam (Cambridge, UK). Blots were exposed to X-ray film for chemiluminescence, following treatment with a West Pico ECL reagent (Thermo Scientific Rockford, IL). Values for the integrated light density of autoradiograms were obtained with Image J NIH software and were employed for the determination of treatment-induced changes in protein levels.

### Detection of cellular ROS

Evaluation of treatment-induced changes in cellular level of ROS was performed by employing CM-H_2_DCFDA as described by Giribaldi et al. (30). Cells were collected with 0.04% crystallized trypsin in Hank's balanced salt solution (HBSS). After washing with HBSS the cells were incubated with freshly prepared 2 μM CM-H_2_DCFDA for 15 minutes in 37°C, washed with HBSS and suspended in HBSS containing 2% FBS. Live cells (~90% of the population) were gated by forward and side scatter and green fluorescence emission (520 nm) from cells illuminated with 492 nm excitation light was measured by fluorescence-activated sorting (FACS) on a Navios flow cytometer (Beckman Coulter) and analyzed using Kaluza analysis software. A minimum of 20,000 cells were acquired for each sample. Staining with 7-aminoactinomycin D (7AAD, eBiosciences, San Diego, CA) verified that the percentage of dead cells within the gated population did not exceed 3%. The data show the distribution of the cells according to their FL1 fluorescence intensities.

### The comet assay

The comet assay was performed with the hOGG1 FLARE^TM^ Assay Kit according to the manufacturer's instructions with slight modifications. The cells were removed by trypsinization with 0.04% crystallized trypsin in HBSS, washed with cold PBS, embedded in agar and layered over the slides. Following incubation with lysis buffer, enzyme reaction buffer and treatment with hOGG1, the cells were further incubated in alkali solution and then subjected to electrophoresis at 21 V for 18 minutes. The cells were stained with 1xSYBR gold and viewed in fluorescent Nikon Eclipse Ti microscope equipped with Nikon Intenslight C-HHGFI camera. Two independent experments were conducted and 100 cells were scored in each sample from two separate experiments. The DNA tails were categorized as either long medium or none. Comets were defined as “long” when their length approximated the diameter of the nucleus from which they originated.

### Wound-healing assay

Cells were seeded into 6-well dishes and grown for 48 hours to 80% confluence. At that time, either TH588 or the vehicle was added to the cells. Four hours later, the bottom of the well was scratched with a perpendicularly placed 1 ml pipette tip. Floating cells were removed, and fresh medium containing either TH588 or the vehicle was added to the wells. Photomicrographs of the wounded monolayers were obtained with an EVOS^®^ FL inverted microscope with a 10x objective at time 0 and every 24 hours thereafter. The width of the scratch was measured by employing the Image J program at 10 different points along the scratch at time 0 and 4 days later at the end of the experiment. It was divided by two to obtain the distance travelled by the cells.

### Matrigel invasion assay

The invasion assay was performed essentially as described by Li et al. [54] The cells were plated in serum-free media onto Matrigel-coated chambers (Nunc^®^, Denmark) and placed into wells containing media that was supplemented with 0.5% FBS. Either TH588 or DMSO was added to the inserted chambers. Following an incubation of 36 hours, the cells from the upper chambers were removed, and the invading cells at the bottom of the filter were stained with crystal violet. Photomicrographs were taken, as described above, for the migration assay, and the cells in each picture were counted with the Image J program. The assay was performed in duplicate, and a total of five randomly chosen fields were counted for each sample.

### Statistical analysis

The significance of the differences between the experimental groups in clonogenic survival was verified by employing the unpaired Student *t* test. P < 0.05 was considered statistically significant.

## Abbreviations

CI: Combination index
DIPG: Diffuse intrinsic pontine glioma
HBSS: Hanks balanced salt solution
HDACi: Histone deacetylase inhibitor
MTH1: Human MutT homolog 1
OGG1: 8-Oxoguanine DNA glycosylase
8-Oxo-dGTP: 8-Oxo-2’-deoxyguanosine-5’-Triphosphate
ROS: Reactive oxygen species
PARPi: Inhibitor of Poly [ADP-ribose] polymerase 1
